# The Risk Factors for Weaning Failure of Mechanically Ventilated Patients With COVID-19: A Retrospective Study in National Medical Team Work

**DOI:** 10.3389/fmed.2021.678157

**Published:** 2021-08-31

**Authors:** Hua Zhao, Longxiang Su, Xin Ding, Huan Chen, Hongmin Zhang, Jinglan Wang, Yun Long, Xiang Zhou, Shuyang Zhang

**Affiliations:** ^1^Department of Critical Care Medicine, Peking Union Medical College Hospital, Chinese Academy of Medical Sciences, Beijing, China; ^2^Department of Respiratory, Peking Union Medical College Hospital, Chinese Academy of Medical Sciences, Beijing, China; ^3^Department of Cardiology, Peking Union Medical College Hospital, Chinese Academy of Medical Sciences, Beijing, China

**Keywords:** weaning failure, mechanical ventilation, COVID-19 pneumonia, risk factors, prognosis

## Abstract

**Purpose:** This study aimed to describe the clinical and laboratory characteristics and the parameters of the respiratory mechanics of mechanically ventilated patients with confirmed COVID-19 pneumonia and to clarify the risk or protective factors for weaning failure.

**Methods:** Patients diagnosed with COVID-19 pneumonia were selected from the special intensive care unit (ICU) of the Sino-French New City Branch of Tong Ji Hospital, Wuhan, and treated by the National Medical Team Work. They were divided into successful weaning (SW) group (*N* = 15) and unsuccessful weaning (USW) group (*N* = 18) according to the prognosis. Information of these patients was analyzed.

**Results:** There were 33 patients included in this study. Patients in the USW group were associated with a poor outcome; the 28-day mortality rate was higher than in the SW group (86.7 vs. 16.7% *p* < 0.001). By comparison, we found that the initial plateau pressure (Pplat) and driving pressure (DP) of the USW group were higher and that compliance was lower than that of the SW group, but there was no difference between positive end-expiratory pressure (PEEP), partial pressure of carbon dioxide (PCO_2_), and the ratio of partial pressure arterial oxygen and fraction of inspired oxygen (P/F ratio). Comparing the worst respiratory mechanics parameters of the two groups, the results of the Pplat, DP, compliance, and PEEP were the same as the initial data. The PCO_2_ of the USW group was higher, while the P/F ratio was lower. A logistic regression analysis suggested that higher Pplat might be an independent risk factor and that higher compliance and lower DP might be protective factors for weaning failure of invasive mechanically ventilated patients with COVID-19 pneumonia.

**Conclusions:** Patients with USW were associated with a poor outcome, higher Pplat might be a risk factor, and a higher compliance and a lower DP might be protective factors for the weaning failure of ventilated COVID-19 patients. Mechanical ventilation settings will affect the patient's prognosis.

## Introduction

The outbreak of COVID-19 disease caused by the novel coronavirus (SARS-CoV-2) has been a worldwide pandemic problem and resulted in thousands of death (1). Its morbidity and mortality are much higher than those of other viral pneumonia. About 15–20% of suspected and confirmed patients developed dyspnea and severe hypoxemia (2); since no specialized medication to treat SARS-CoV-2 infection has been identified at this time, mechanical ventilation is the main supportive treatment for critically ill patients, especially invasive mechanical ventilation. Whether the ventilated patients can wean successfully is a key factor related to the patient's outcome. The mortality of ventilated patients with COVID-19 pneumonia was high (86.3% 19/22) in an observational study from a single center, the Jinyintan Hospital (a temporarily designated center for critically ill patients with COVID-19), Wuhan, China (3). The parameters of the respiratory mechanics, especially plateau pressure (Pplat), transpulmonary pressure (Ptp), or driving pressure (DP), were the major risk factors for the acute respiratory distress syndrome (ARDS) patients (4). Recent studies showed that the DP was high, respiratory system compliance was low, and hypercapnia was common in the ventilated patients with COVID-19 while using low tidal volume ventilation (5, 6), but the sample size was small, and the risk factors for the weaning failure of the ventilated COVID-19 patients were not described.

We aim to describe the clinical and laboratory characteristics, and the parameters of the respiratory mechanics of mechanically ventilated patients with confirmed COVID-19 pneumonia, and to clarify the risk or protective factors for weaning failure. We hope our study findings will inform on the global fight against the COVID-19 disease.

## Materials and Methods

### Study Design and Patient Enrollment

This study was a retrospective clinical study at the special intensive care unit (ICU) of the Sino-French New City Branch of Tong Ji Hospital, Wuhan. All subjects were selected from the population of inpatients hospitalized between February 2020 and March 2020 in the departments mentioned above. This study was approved by the ethics review board of PUMCH (ZS-2332).

### Inclusion and Exclusion Criteria

The following inclusion criterion was used: patients with diagnosed COVID-19 by nucleic acid detection and imaging evidence based on the Novel Coronavirus Pneumonia Diagnosis and Treatment Program (Version 7) released by China Health Commission. Patients were excluded from the study if they met the following criteria: (i) under 18 years of age and (ii) died within 48 h of admittance to the ICU. Patients who satisfied the inclusion criteria were divided into a successful weaning (SW) group and an unsuccessful weaning (USW) group in accordance with the outcome during the treatment. USW is defined as either the failure of spontaneous breathing trial (SBT) or the need for invasive mechanical ventilation or reintubation within 48 h following weaning or extubation in our study (7). Failure of SBT is defined by objective indices of failure, such as tachypnea, tachycardia, hypertension, hypotension, hypoxemia or acidosis, and arrhythmia (8).

### Treatment

Based on the respiratory mechanics features and current medical conditions, we performed a ventilation strategy. (1) Initial application of lung protection ventilation strategy: low tidal volume (VT) ventilation (VT 4–6 ml/kg of predicted body weight), the target Pplat was set at lower than 30 cmH_2_O, a higher positive end-expiratory pressure (PEEP) strategy ≥10 cmH_2_O, usage of sedative and analgesic drugs and neuromuscular blocking agents (NMBAs). (2) Prone position ventilation (PPV) was performed when the ratio of partial pressure arterial oxygen and fraction of inspired oxygen (P/F ratio) was <150 and was performed at the physicians' discretion. (3) If refractory hypercapnia exists, evaluate the recruitability; a higher level of PEEP and recruitment maneuvers may help to reduce hypercapnia and acidosis in patients who were recruited by reducing physiologic dead space and shunt. (4) In those who had no potential for recruitment, increase the respiratory rate (RR) to 30–35 bpm, lower the PEEP, increase VT to 8 ml/kg, and ensure that the Pplat <30 cmH_2_O and the DP <17 cmH_2_O. (5) Due to shortage of extracorporeal membrane oxygenation (ECMO), extracorporeal carbon dioxide removal (ECCO_2_R) may mitigate hypercapnia. Routine procedures for the hemodynamic therapy and a conservative fluid strategy were performed, and antibiotics were administered intravenously at the discretion of the attending clinician.

### Measurements and Data Collection Involved in this Study

All the data of these involved patients were recorded, including general data, mechanical ventilation data, hemodynamic data, outcome, and other therapies. The general data included the age, gender, Sequential Organ Failure Assessment (SOFA) score, Acute Physiology and Chronic Health Evaluation II (APACHEII) score, Glasgow coma scale (GCS), white blood cells (WBCs), lymphocytes, platelets, total bilirubin (Tbil), creatinine, prothrombin time, fibrinogen (Fbg), D-dimer, procalcitonin (PCT), IL-6, high-sensitivity C-reactive protein (hs-CRP), and high-sensitivity cardiac troponin I (hs-CTNI). In addition, comorbidities (hypertension, diabetes, coronary heart disease, chronic renal insufficiency, chronic underlying lung disease, and cerebrovascular disease), the time from the symptom onset to intubation, and the duration of non-invasive ventilation (NIV)/high-flow oxygen therapy (HFOT) were recorded. The initial and worst mechanical ventilation data included the Pplat, DP, VT, RR, partial pressure of carbon dioxide (PCO_2_), (P/F), and minute ventilation (MV). The initial respiratory mechanics parameters were defined as the value of the first day of intubation or admittance to the ICU. The worst respiratory mechanics parameters were defined as the worst value in the treatment process after ICU admission, including the lowest value of P/F, lung compliance, and the highest values of Pplat, DP, PCO_2_, and PEEP. The hemodynamic data included heart rate (HR), mean arterial pressure (MAP), left ventricular ejection fraction (LVEF), and lactate (lac). The outcome included ventilation-free day (VFD) of 28 days, the length of stay in ICU, 28 days' mortality, and the complications [pneumothorax, ventilator-associated pneumonia (VAP), bloody infection, acute kidney injury (AKI), myocardial injury, coagulopathy, and liver injury]. Acute kidney injury was identified on the basis of serum creatinine. Cardiac injury was diagnosed if the serum concentration of hs-CTNI was above the upper limit of the reference range (>15.6 pg/ml), measured in the laboratory of Tong Ji Hospital. Other therapies included PPV, ECMO, ECCO_2_R, continuous renal replacement therapy (CRRT), and the use of vasoactive drugs.

### Statistical Analysis

Statistical analyses were performed using SPSS 16.0 (SPSS, Chicago, IL, USA), and a *p* < 0.05 was considered significant. Quantitative data with normal distributions are denoted as means ± standard deviation. Student's *t*-test was performed to compare means between the two groups. Quantitative data that were abnormally distributed are denoted as medians (interquartile ranges), and the rank-sum test was performed for these data. Data of unordered categories are denoted as rates, and differences between groups were examined using the chi-squared test or Fisher's exact test. The multi-factor logistic regression was used to estimate differences between the two groups and to explore the risk factors.

## Results

### The Demographic and Clinical Characteristics of the Patients Involved in this Study

**Figure 1:**
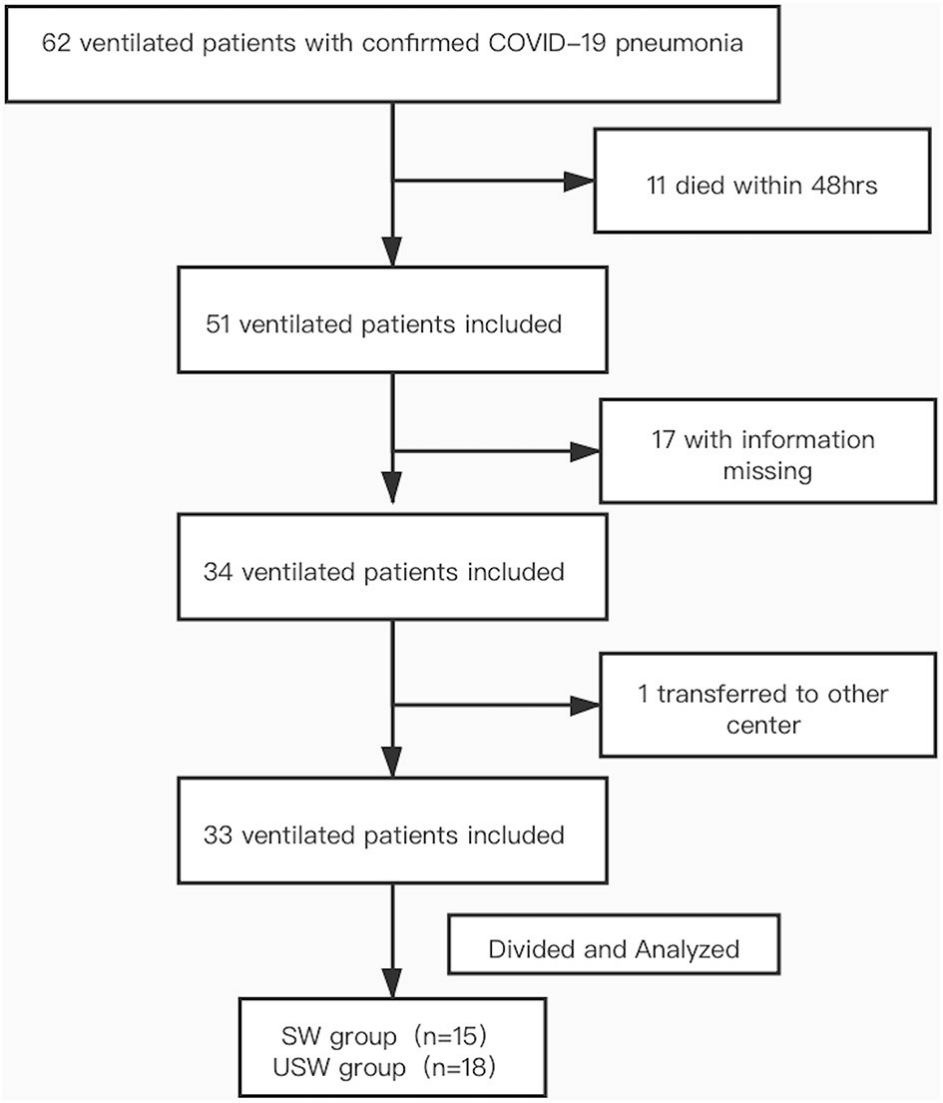
Flowchart.

By March 2020, 62 ventilated patients with confirmed COVID-19 pneumonia had been admitted to the special ICU of the Sino-French New City Branch of Tong Ji Hospital, of whom 29 were excluded (11 died within 48 h of ICU admission, 17 with missed information, and one transferred to another center). There were 33 patients involved in this study, including 15 cases of SW group and 18 cases of USW group (as shown in Figure 1). The demographic and clinical characteristics of the included patients are summarized in Table 1. There was no difference between the two groups in term of age (*p* = 0.977), gender (*p* = 0.948), SOFA score (*p* = 0.093), and APACHE II score (*p* = 0.229). Patients in the USW group were associated with a poor outcome, the 28-day mortality rate was higher than in the SW group (86.7 vs. 16.7% *p* < 0.001), and VFD of 28 days and ICU stays were lower than in the SW group (*p* = 0.022, 0.001). The PCT, IL-6, and hs-CRP were higher in the USW group; *p*-values were 0.018, 0.009, and 0.004, respectively. The comorbidities hypertension, diabetes, and coronary heart disease also had significant differences. In addition, there were no significant differences in terms of hemodynamic parameters, interventions, the time before intubation, and the time for NIV/HFOT between the two groups.

**Table 1:** The Baseline characteristics and outcomes of the SW group vs. USW group.

	**SW group *N* = 15**	**USW group *N* = 18**	***P*-value**
Age, years	62.3 ± 13.6	62.2 ± 8.0	0.977
Gender			0.948
Male	9(60)	11(61.1)	
Female	4(40)	7(38.9)	
SOFA score	8(3,14)	10(5,17)	0.093
APACHE II score	16(12,22)	18(13,28)	0.229
GCS	13(5,15)	12(8,15)	0.556
PO2/FiO2	175(75,220)	110(37,240)	0.093
HR (bpm)	95(63,130)	102(70,134)	0.708
MAP(mmHg)	80(70,95)	86(55,102)	0.290
Time before Intubation (days) median (IQR)	15(3,28)	17.5(7,41)	0.093
Time for NIV/HFOT (days) median (IQR)	3(0,9)	4(0,28)	0.135
**Comorbidities n (%)**			
Hypertension	1(6.7)	9(50)	<0.001
Diabetes	6(40)	1(5.6)	0.016
Coronary heart disease	11(73.3)	2(11.1)	<0.001
Chronic renal insufficiency	0(0)	0(0)	1
Chronic underlying lung disease	3(20)	7(38.9)	0.24
Cerebrovascular disease	4(26.7)	4(22.2)	0.767
**Laboratory examination (Initial)**			
White blood cell count ×10^9^/L)	10.5(3.9,26.7)	12.4 (6.3,35.6)	0.442
Lymphocyte count(×10^9^/L)	0.56(0.27,1.86)	0.55(0.24,0.96)	0.817
Platelet count(×10^12^/L)	172(79,331)	151(28,459)	0.789
Total bilirubin (μmol/L)	9.1(4.1,19.7)	11.4(3.8,42.2)	0.274
Creatinine (μmol/L)	64(35,266)	64(40,427)	0.885
Prothrombin time (s)	15.3(13.5,17.6)	16.1(13.5,45)	0.190
Fibrinogen (g/L)	4.3(1.7,7.0)	5.3(0.6,8.8)	0.656
D-dimmer(μg/mL,FEU)	4.2(0.7,21)	20.1(2.72,21)	0.178
PCT (ng/mL)	0.7(0.08,4.5)	2.7(0.04,24.8)	0.018
IL-6 (pg/mL)	96(10,5000)	2287(18,5000)	0.009
Hypersensitive CRP (mg/L)	122(50,314)	244(21,320)	0.004
Hypersensiyive troponin I(pg/L)	84(12,1873)	229(16,7762)	0.117
PCO2(mmHg)	49(42,53)	49(36,110)	0.735
PH	7.33(7.11,7.42)	7.29(6.82,7.52)	0.901
Lactate(mmol/L)	2.3(1.5,4.3)	1.9(1.0,6.5)	0.135
**Interventions**			
Prone position ventilation *n* (%)	11(73.3)	13(72.2)	0.627
ECMO *n* (%)	1(6.7)	2(11.1)	0.570
ECCO2R *n* (%)	2(13.3)	4(22.2)	0.51
Vasoactive drugs *n* (%)	10(66.7)	15(83.3)	0.240
CRRT *n* (%)	4(26.7)	5(27.8)	0.627
**Outcomes**			
ICU stay (days), median (IQR)	37(8,65)	14(4,51)	0.001
28d mortality (%)	16.7	86.7	<0.001
Duration of non-ventilation within 28d (days), median(IQR)	0(0,23)	0(0,0)	0.022

*SOFA score, Sequential Organ Failure Assessment score; APACHE II score, Acute Physiology and Chronic Health Evaluation II score; GSC, Glasgow coma scale; HR, heart rate; MAP, mean atrial pressure; NIV, non-invasive ventilation; HFOT, high flow oxygen therapy; PO2/FIO2, the ratio of partial pressure arterial oxygen and fraction of inspired oxygen; PCO2, partial pressure of carbon dioxide; PH, potential of hydrogen; ECMO, extracorporeal membrane oxygenation; ECCO2R, extracorporeal carbon-dioxide removal; Prone position ventilation, Received at least one session of prone positioning; CRRT, continuous renal replacement therapy*.

### The Mechanical Ventilation Parameters at the Initial and Worst Stages

**Figure 2:**
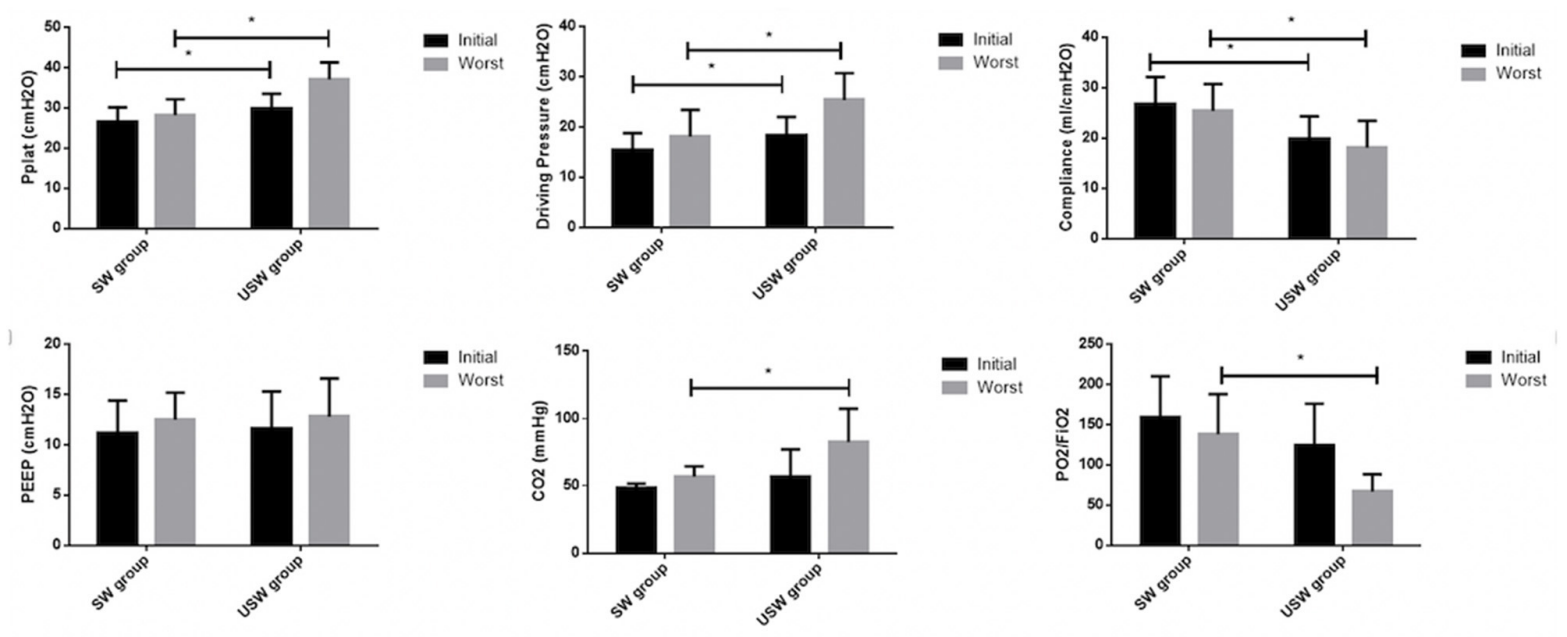
The mechanical ventilation parameter comparison between successful weaning (SW) and unsuccessful weaning (USW). Initial, the first day of intubation or admittance to the intensive care unit (ICU); Worst, the worst in the observation days; PEEP, positive end-expiratory pressure; Pplat, plateau pressure; PCO_2_, partial pressure of carbon dioxide; PaO_2_/FiO_2_, the ratio of partial pressure arterial oxygen and fraction of inspired oxygen. *means the *p*-value <0.05 between two groups.

We compared the mechanical ventilation parameters (initial) of the first day of intubation or admittance to the ICU and those of the worst stage in the course of treatment. By comparison, we found that the initial Pplat and DP of the USW group were higher and that compliance was lower than that of the SW group, but there was no difference between PEEP, PCO_2_, and P/F ratio. Comparing the worst respiratory mechanics parameters of the two groups, the results of the Pplat, DP, compliance, and PEEP were same as the initial data. The PCO_2_ of the USW group was higher, while the P/F was lower (Figure 2).

### Complication Comparison for Successful Weaning Vs. Unsuccessful Weaning

The USW group had more myocardial injury, coagulopathy, and liver injury than the SW group as shown in Table 2 (*p*-values were 0.001, 0.002, and 0.008 respectively); there was no difference in the incidence of other complications (*p* > 0.05).

**Table 2:** Complication comparison for successful weaning (SW) vs. unsuccessful weaning (USW).

	**SW group**	**USW group**	***P*** **-value**
Pneumothorax *n*(%)	0(0)	4(22.2)	0.051
VAP *n*(%)	11(73.3)	12(66.7)	0.678
Blood infection *n*(%)	4(26.7)	3(16.7)	0.484
AKI *n*(%)	6(42.9)	8(44.4)	0.928
Myocardial injury *n*(%)	7(53.8)	18(100)	0.001
Coagulopathy *n*(%)	5(35.7)	16(88.9)	0.002
Liver injury *n*(%)	0(0)	7(38.9)	0.008

*VAP, ventilator-associated pneumonia; AKI, acute kidney injury*.

### Risk Factors for Weaning Failure of Mechanically Ventilated Patient

The Table 3 showed that the Pplat was a risk factor for unsuccessful weaning (OR = 1.81, 95% CI 0.951–3.476), Low driving pressure and high compliance were protective factors, The OR of low driving pressure and high compliance were 0.551 (95% CI, 0.26–1.1 67) and 0.55 (95% CI, 0.274–1.1 02), respectively.

**Table 3:** Multifactor logistic regression analysis for unsuccessful weaning risk factors.

**Variables**	**B**	**SE**	**Wald's coefficient**	**OR**	**95% CI for OR**		***P*** **-value**
**Lower**	**Upper**
Pplat(cmH_2_O)	0.598	0.331	3.264	1.81	0.951	3.476	0.027
DP(cmH_2_O)	−0.596	0.383	2.421	0.551	0.26	1.167	0.029
compliance	0.598	0.355	2.839	0.55	0.274	1.102	0.03

*Pplat, plateau pressure; DP, Driving pressure*.

## Discussion

This retrospective study provided a detailed analysis of invasive mechanical ventilation of COVID-19 patients and explored the risk factors for the weaning failure in one tertiary center of Wuhan, China. Our results indicated that patients in the USW group were associated with a poor outcome and more complications. Furthermore, we found that high Pplat was a risk factor for USW and that low DP and high compliance were protective factors.

In this single-center observational study, the mortality of ventilated COVID-19 patients was high compared with that of other reports at 25–50% (9–11); we assumed that the respiratory mechanics characteristics of our patients were not the same as those of the two phenotypes reported by Gattinoni et al. (12). Weaning from mechanical ventilation is an individualized process in which a gentle balance between respiratory system load and capacity must be achieved (13). Our result showed that patients with weaning failure had a poor outcome and more complications, which was similar to the previous study (14–16). A multicenter prospective study also found that the mortality rate of patients with successful liberation from MV of 28 days was lower than that of the unsuccessful group (0 vs. 62.4%) (14). Our findings indicate the importance of weaning failure in the management of COVID-19; whether the ventilated patients can be weaned successfully is a key factor related to the patient's mortality rate.

We observed that the USW group had higher Pplat, DP, and PCO_2_ and lower VT, P/F ratio, and compliance than the SW group (*p* < 0.05). Pplat was the risk factor for USW, and low DP and high compliance are protective factors in this study. Our findings were not consistent with those of other centers. Oadya found no statistically significant correlation between patients' characteristics and the weaning failure (15), and a study from Japan observed a decreasing trend in respiratory static compliance despite the higher PEEP setting after day 5 and a higher ventilatory ratio in patients with prolonged MV than in those with early liberation (16). In the landmark ARDS Network trial, long-term mortality improved when VT was limited to an average of 6 ml/kg of predicted body weight and Pplat to <30 cmH_2_O (17). Pplat is the sum of PEEP and DP. The mechanical effects of high PEEP depend on lung recruitability (18) and are harmful (hyperinflation of previously opened alveoli) for our patients with non-potential for recruitment. DP corresponds to the elastic pressure swing; excessive DP increases the risk of VT-induced strain and is associated with higher mortality (19, 20). In the PRoVENT-COVID study, the median of DP was 14 cmH_2_O (19). Actually, recent data (21) have demonstrated that there is no safe upper limit for DP; the slope of the relationship between DP and mortality appears to be positive even at DP below 14 cmH_2_O, suggesting that patient outcomes may be improved with the decreasing DP. The SATICOVID study also showed that DP was strongly associated with mortality (22). Regardless of whether it is due to the high peep or DP, a high Pplat (close to 30 cmH_2_O) is an important cause for alarm for clinicians. In prior studies, respiratory compliance has not been an independent factor for weaning failure when it was measured early in the ARDS (23, 24). Compliance <40 ml/cmH_2_O has been recently proposed to identify a more severe phenotype of COVID-19 (25). In Gamberini's study, the results showed that compliance <40 ml/cmH_2_O was independently associated with both prolonged mechanical ventilation and mortality (15). Our findings may be explained in part by using the worst compliance. The worst compliance of USW group patients was only 11.67 ± 4.51 ml/cmH_2_O, which were lower than that reported in other centers. PPV had been used to COVID-19 pneumonia in our patients (73.3% in the SW group and 72.2% in the USW group). There was no difference between the two groups. The reason may be that the pathophysiological phenotypes of the two groups were different; PPV has no obvious effectiveness in the USW group. These findings reminded us that in early implementation of lung protection strategies, lowering plateau pressure and DP is important to avoid lung injury in COVID-19 patients. As the sample size with extracorporeal support was too small due to shortage of resources, there was no difference between the two groups in our study. We still recommend that if conventional methods do not work, special respiratory therapy such as ECCO_2_R and ECMO should be performed as soon as possible.

This study has several limitations. First, this study was conducted at a single-center hospital with limited sample size. There may also be a selection bias when identifying factors that influence the clinical outcomes. A larger cohort study of ventilated patients with COVID-19 pneumonia would help to further define the clinical characteristics and risk factors of the disease. Second, some patients failed to enroll because specific information was missing. However, the 33 patients we enrolled had all the detailed results of complete respiratory mechanics monitoring and dynamic records. This is very precious. Third, this is a retrospective study; the data in this study permit a preliminary assessment of the outcomes of critically ill patients with SARS-CoV-2 pneumonia. Further studies are still needed.

## Conclusion

Based upon an analysis of the data from mechanically ventilated COVID-19 patients, it can be observed that patients with weaning failure were associated with a poor outcome and more complications. A higher Pplat might be a risk factor; a higher compliance and a lower DP might be protective factors for the weaning failure of ventilated COVID-19 patients. Early implementation of lung protective strategies and lower plateau pressure and DP are important to avoid lung injury in COVID-19 patients.

## Data Availability Statement

The raw data supporting the conclusions of this article will be made available by the authors, without undue reservation.

## Ethics Statement

The studies involving human participants were reviewed and approved by Ethics review board of PUMCH (ZS-2332). Written informed consent for participation was not required for this study in accordance with the national legislation and the institutional requirements. Written informed consent was obtained from the individual(s) for the publication of any potentially identifiable images or data included in this article.

## Author Contributions

XZ and SZ: study conceptualization and writing—review and editing. HZ and LS: study design, formal analysis and investigation, and writing—original draft preparation. HZ, XD, HC, HZ, JW, and SZ: methodology. All authors commented on the previous version of the manuscript and have read and approved the final manuscript.

## Conflict of Interest

The authors declare that the research was conducted in the absence of any commercial or financial relationships that could be construed as a potential conflict of interest.

## Publisher's Note

All claims expressed in this article are solely those of the authors and do not necessarily represent those of their affiliated organizations, or those of the publisher, the editors and the reviewers. Any product that may be evaluated in this article, or claim that may be made by its manufacturer, is not guaranteed or endorsed by the publisher.
